# Supplementary material for: Threefold coordinated germanium in a GeO2 melt
Source: Nat Commun. 2023 Nov 2;14:7008. doi: 10.1038/s41467-023-42890-3 (PMC10622558; doi:10.1038/s41467-023-42890-3)
Supplement: Supplementary file 1 — Supplementary Information [file 41467_2023_42890_MOESM1_ESM.pdf]

Supplementary Information

Threefold coordinated germanium in a GeO_2 melt

Wan et al.

This PDF file includes

- Supplementary Figures 1 to 4
- Supplementary Tables 1 to 3
- Supplementary Note
- Supplementary References

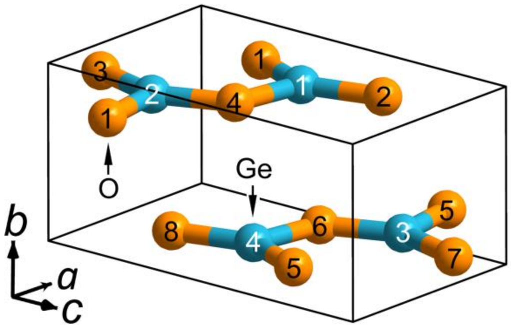

Supplementary Fig. 1 | Structural model of the $[GeOØ_2]_n$ chain. Blue and orange spheres represent germanium and oxygen atoms, respectively (the same below). Four GeO_2 formulae are placed into a periodic, orthogonal unit cell with dimensions of a = 4.85 Å, b = 3.82 Å and c = 8.15 Å. The unit cell has the *Pnma* symmetry (space group No.62). The germanium and oxygen atoms construct two $[GeOØ_2]_n$ chains extending along the *a*-axis.

Supplementary Table 1 | Atomic coordinates for the $[GeOØ_2]_n$ chain model

Atom	*x/a*	*y/b*	*z/c*
Ge1	0.916	3/4	0.369
Ge2	0.416	3/4	0.131
Ge3	0.584	1/4	0.869
Ge4	0.084	1/4	0.631
O1	0.056	3/4	1/6
O2	0.017	3/4	0.587
O3	0.517	3/4	0.913
O4	0.556	3/4	1/3
O5	0.944	1/4	5/6
O6	0.444	1/4	2/3
O7	0.483	1/4	0.087
O8	0.983	1/4	0.413

Supplementary Table 2 | Thirty-six vibrational bands of the $[GeOØ_2]_n$ chain and their computational frequencies and Raman/IR intensities

No.	ω (cm^{-1})	IR intensity (km/mol)	Raman intensity ($Å^4$/amu)	No.	ω (cm^{-1})	IR intensity (km/mol)	Raman intensity ($Å^4$/amu)
1	−0.0	/	/	19	358.0	/	0.04
2	−0.0	/	/	20	382.7	17.3	/
3	−0.0	/	/	21	412.8	/	/
4	96.4	/	/	22	446.7	1459.5	/
5	107.4	/	4.6	23	514.9	338.9	/
6	159.6	/	0.4	24	545.2	/	346.0
7	164.5	/	0.04	25	598.7	486.4	/
8	169.3	/	0.8	26	636.5	/	3.5
9	182.1	0.1	/	27	646.0	1247.4	/
10	206.9	/	31.0	28	713.0	/	1.2
11	216.5	2213.0	/	29	718.3	/	84.8
12	220.0	33.8	/	30	738.9	50.7	/
13	243.2	/	/	31	795.2	/	13.4
14	250.0	/	0.7	32	795.8	/	2.6
15	258.0	/	2.2	33	833.9	1403.3	/
16	268.6	/	0.9	34	837.9	/	21.1
17	333.0	/	79.7	35	887.0	511.7	/
18	350.3	391.7	/	36	937.2	/	2.0

Supplementary Table 3 | Twenty-seven vibrational bands of quartz-type GeO_2 and their computational frequencies and Raman/IR intensities

No.	ω (cm^{-1})	IR intensity (km/mol)	Raman intensity ($Å^4$/amu)	No.	ω (cm^{-1})	IR intensity (km/mol)	Raman intensity ($Å^4$/amu)
1	−0.0	/	/	15	347.7	37.6	/
2	−0.0	/	/	16	448.2	/	323.9
3	−0.0	/	/	17	516.7	114.9	15.1
4	118.7	0.8	1.2	18	516.7	114.9	12.1
5	118.7	0.8	0.9	19	535.4	462.7	/
6	163.9	/	9.7	20	584.2	106.9	3.9
7	197.2	5.1	0.5	21	584.2	106.9	3.6
8	197.2	5.1	0.5	22	861.9	1204.3	4.6
9	231.2	180.0	/	23	861.9	1204.3	4.7
10	243.2	145.4	1.6	24	878.4	1250.8	/
11	243.2	145.4	2.2	25	885.8	/	12.2
12	252.21	/	8.3	26	968.7	11.8	2.5
13	317.3	174.1	0.7	27	968.7	11.8	2.0
14	317.3	174.1	0.8				

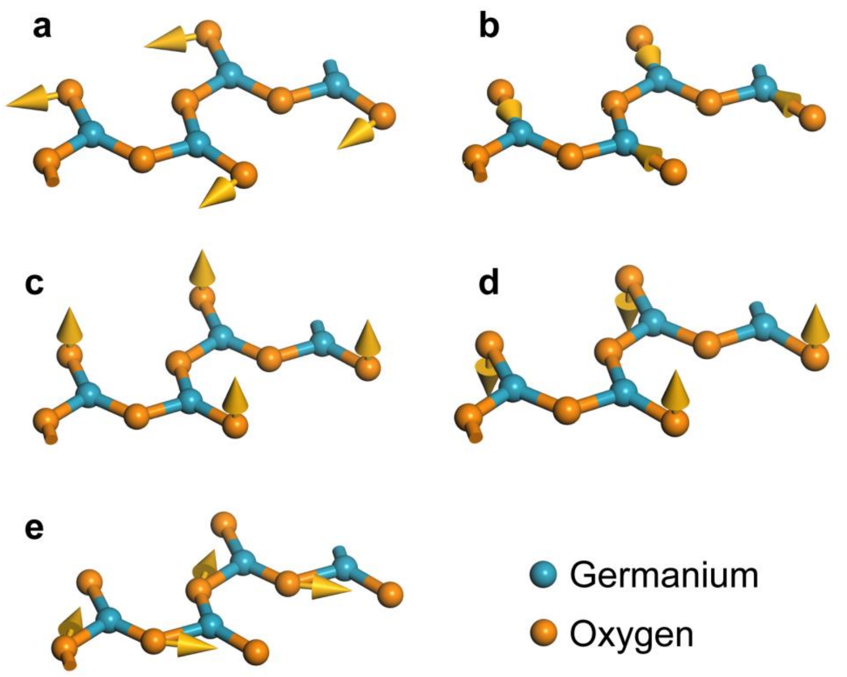

Supplementary Fig. 2 | Atomic motions for five characteristic Raman bands of the $[GeOØ_2]_n$ chain, located at (a) 207 cm^{-1}, (b) 718 cm^{-1}, (c) 795 cm^{-1}, (d) 796 cm^{-1} and (e) 838 cm^{-1}.

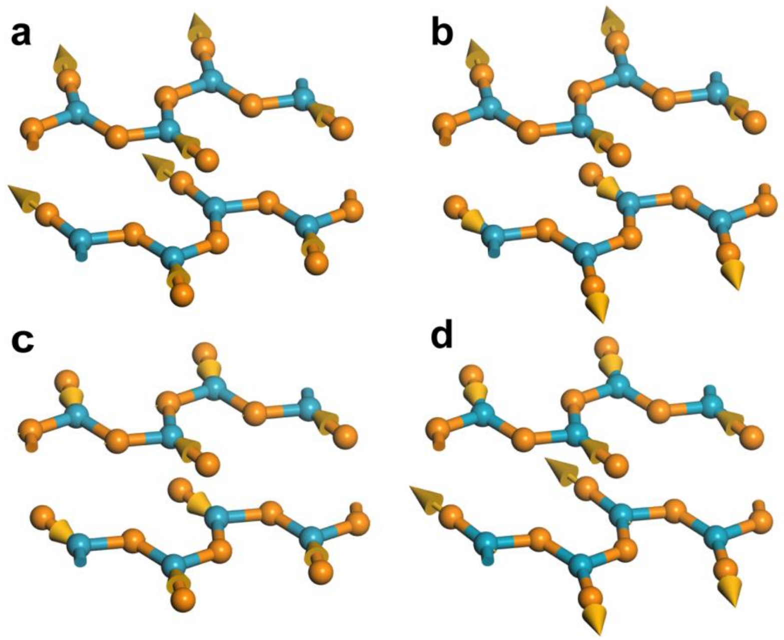

Supplementary Fig. 3 | Four Ge−O stretching modes of the $[GeOØ_2]_n$ chain, located at (a) 646 cm^{-1}, (b) 713 cm^{-1}, (c) 718 cm^{-1}, (d) 739 cm^{-1}. The 713 and 718 cm^{-1} bands are Raman active.

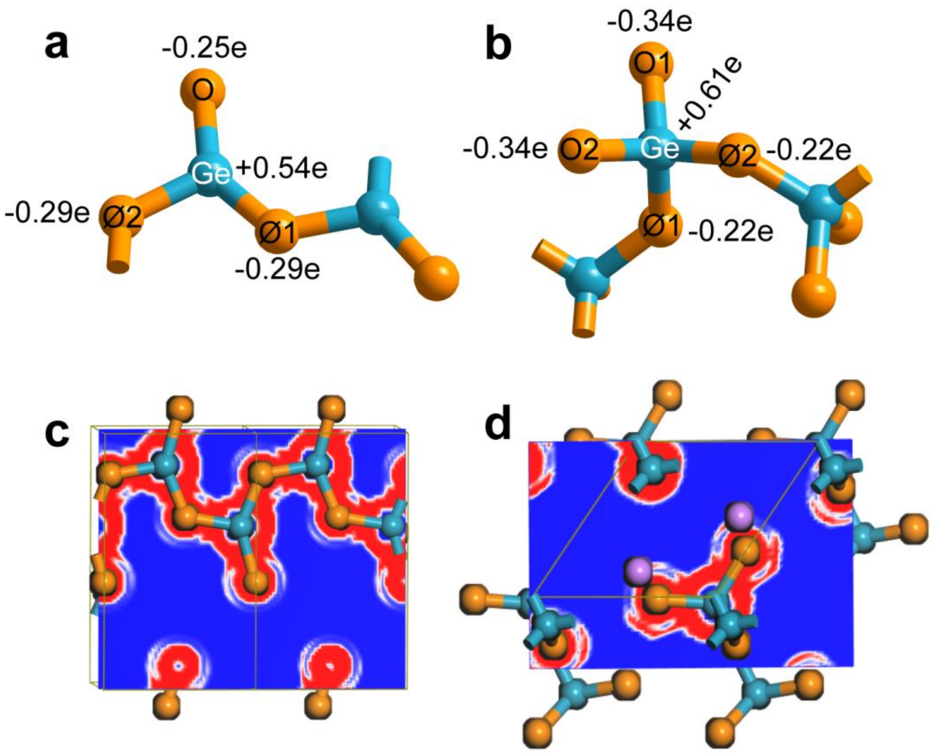

Supplementary Fig. 4 | Hirshfeld atomic charges and valence ELFs of the $[GeOØ_2]_n$ and the $[GeO_2Ø_2^{2-}]_n$ chains. **a** Hirshfeld atomic charges of the $[GeOØ_2]_n$ chain. **b** Hirshfeld atomic charges of the $[GeO_2Ø_2^{2-}]_n$ chain. **c** Valence ELF of the $[GeOØ_2]_n$ chain. **d** Valence ELF of the $[GeO_2Ø_2^{2-}]_n$ chain. Purple spheres represent lithium atoms. The maps are along the planes on which the Ge−O bonds lie.

Supplementary Note 1

The Li_2GeO_3 crystal structure, reported by Völlenkle, is built up of Li^+ cations and $[GeO_2Ø_2^{2-}]_n$ chains (Supplementary Fig. 4b and 4d)[1]. The structure was adopted as the model for analysis of the Hirshfeld atomic charges and valence ELF of the $[GeO_2Ø_2^{2-}]_n$ chain. Except the *k*-point grid (3 × 3 × 3), all of the computational parameters were the same as those for the $[GeOØ_2]_n$ chain.

For the $[GeOØ_2]_n$ chain, the Hirshfeld charges of Ge, non-bridging O and bridging O are +0.54 e, −0.25 e and −0.29 e, respectively (Supplementary Fig. 4a). For the $[GeO_2Ø_2^{2-}]_n$ chain, the Hirshfeld charges of Ge, non-bridging O and bridging O are +0.61 e, −0.34 e and −0.22 e, respectively (Supplementary Fig. 4b). The strengths of the Ge–O bonds in the two chains are evaluated by the valence ELFs. The electron pairs around the Ge–O bonds in the $[GeOØ_2]_n$ chain (Supplementary Fig. 4c) are less than that in the $[GeØ_2O_2^{2-}]_n$ chain (Supplementary Fig. 4d), indicating that the Ge–O bonds in the $[GeOØ_2]_n$ chains are weaker than that in the $[GeO_2Ø_2^{2-}]_n$ chain. The conclusion is consistent with the Ge–O bond length data (1.84 Å in the $[GeOØ_2]_n$ chain and 1.73 Å in the $[GeO_2Ø_2^{2-}]_n$ chain).

Supplementary References

1. Völlenkle, H. Verfeinerung der Kristallstructuren von Li_2SiO_3 und Li_2GeO_3. *Z. Kristallogr. - Cryst. Mater.* **154,** 77−81 (1981)
